# Retrospective Study Comparing Treatment Outcomes in Obstetric Patients With Iron Deficiency Anemia Treated With and Without Intravenous Ferric Carboxymaltose

**DOI:** 10.7759/cureus.55713

**Published:** 2024-03-07

**Authors:** Mingyue Li, Ann Wright, Asmira M Rahim, Kok Hian Tan, Shephali Tagore

**Affiliations:** 1 Obstetrics and Gynaecology, KK Women's and Children's Hospital, Singapore, SGP; 2 Maternal Fetal Medicine, KK Women's and Children's Hospital, Singapore, SGP

**Keywords:** ferinject, iron deficiency, pregnancy, anemia, obstetrics

## Abstract

Introduction

Iron deficiency anemia is associated with an increased risk of adverse maternal and perinatal outcomes. Intravenous iron preparation containing ferric carboxymaltose has been shown to be a safe and effective way of increasing hemoglobin (Hb) and mean corpuscular volume (MCV) levels and reducing the need for blood transfusion. In our center, it used to be given as an inpatient procedure because of the risks of potential drug reactions. In 2021, we initiated the administration of intravenous ferric carboxymaltose as an outpatient procedure. We compared the outcomes of patients between 2021 and 2023 after the initiation of outpatient administration of intravenous ferric carboxymaltose in 127 obstetric patients with iron deficiency anemia in the second and third trimesters.

Methods

In this study conducted in a large maternity unit in Singapore between 2021 to 2023, we compared the changes in maternal hematological parameters among obstetric patients with iron deficiency anemia presenting to the day care unit in the second or third trimester with a Hb level of <8 g/dl treated with a single dose of ferric carboxymaltose injection (Ferinject) against a control group who were referred for treatment but defaulted on and declined treatment.

Results

Ferric carboxymaltose significantly increased the Hb and MCV levels at delivery in obstetric patients with iron deficiency. The mean Hb at delivery was 10.8 g/dL in the case group compared to 8.8 g/dL in the control group. The percentage of patients with Hb ≥10.0 g/dL was 73.4% in the case group compared to 27.8% in the control group. The incidence of adverse side effects was low and mild (2/127; 1.6%). None of the patients received were hospitalized because of ferric carboxymaltose.

Conclusion

A single injection dose of ferric carboxymaltose as an outpatient antenatal procedure was easily administered and well tolerated. Obstetric patients with iron deficiency anemia who received intravenous ferric carboxymaltose had a significantly higher level of Hb than those who did not.

## Introduction

Anemia in pregnancy is defined as first-trimester hemoglobin (Hb) less than 11.0 g/dL, second-/third-trimester Hb less than 10.5 g/dL, and postpartum Hb less than 10.0 g/dL according to guidelines from the Royal College of Obstetricians and Gynaecologists (RCOG), in line with British Committee for Standards in Haematology (BCSH) guidelines [1]. Microcytic, hypochromic anemia in pregnancy is common. Iron deficiency in combination with the normal physiological circulatory changes of pregnancy, as well as noncompliance to treatment, are the usual causes, but hemoglobinopathies, chronic losses from, for example, helminthic infections, malaria, blood dyscrasias, and other nutritional deficiencies, need to be excluded.

Iron deficiency is one of the most common causes of anemia among the obstetric population, complicating nearly 50% of pregnancies globally [2], affecting more than 56 million women, with the majority from Asia [3]. Iron deficiency anemia is associated with an increased risk of adverse maternal and perinatal outcomes including preterm birth, fetal growth restriction, and postpartum infections [4]. For the mother, as well as a higher level of fatigue reducing work capacity, it has been associated with a higher rate of pregnancy complications including preterm labor and delivery [5], preeclampsia [6], abruption [7], and increased vulnerability to postpartum hemorrhage. Iron deficiency more than doubles the rate of infant mortality [8] and increases maternal mortality risk by making the mother more prone to cardiac failure. Iron is essential for cell function, with roles in oxygen and electron transport and enzyme activity, and adequate levels are particularly important in situations where cell turnover is high, most notably in the developing fetal brain. Maternal iron deficiency has been associated with the recognition, memory, and processing problems in infancy, which can lead to poor bonding and childhood autism and schizophrenia [9,10].

Early diagnosis and correction are necessary with particular focus on mothers in high-risk groups, including those at the extremes of reproductive age and in lower socioeconomic groups. Low body mass index (BMI), high parity, and late antenatal booking are also risk factors for maternal iron deficiency anemia [11].

Diagnosis is centered around a full blood count (FBC) and film with an iron panel including ferritin, Hb electrophoresis for thalassemia, and exclusion of vitamin B12 and folate deficiencies. Serum ferritin is the most sensitive screening test for iron stores [12]. According to the World Health Organization, serum ferritin lower than 15 μg/L in pregnant women is the recommended cutoff value to define iron deficiency [13]. According to guidelines by the British Committee for Standards in Haematology, treatment should be considered when serum ferritin levels fall below 30 μg/L, as this indicates early iron depletion, which may worsen without adequate treatment [14]. Although the mechanisms for iron availability have not been fully elucidated and therefore optimal dosage and frequency schedules remain unknown, the current mainstay of treatment for women who are not allergic is iron supplementation either orally or parenterally depending on the Hb level and gestational age to improve maternal and perinatal outcomes and reduce the need for blood transfusion, although in cases of a very low Hb blood transfusion may be obligatory (Hb<5 g/dl at less than 36 weeks and <6 g/dl at more than 36 weeks in the absence of signs of decompensation, sickling, severe malaria, serious bacterial infection or pre-existing heart disease).

Intravenous formulation containing the iron carbohydrate carboxymaltose (e.g., Ferinject) does not have the allergenic properties of dextran-containing intravenous iron products. It contains 1,000 mg of iron per 20 mL of infusion. It has high bioavailability and has been used in patients with non-absorptive bowel disorders [15] and cardiac failure [16] to improve Hb levels. Moreover, it has been shown to be a safe and effective form of treatment for maternal iron deficiency anemia [17] and is potentially useful for pregnant women with anemia with or without symptoms at later gestations for whom there may be inadequate time to correct orally and among whom its use has been increasing.

We aim to study the effectiveness of intravenous ferrous carboxymaltose in obstetric patients with iron deficiency anemia and its effects on outcomes at delivery and the safety of giving it as an outpatient procedure.

## Materials and methods

KK Women’s and Children’s Hospital (KKH) is a large tertiary maternity unit in Singapore with 11,000 to 12,000 deliveries yearly. It offers a comprehensive package of obstetric care for both low- and high-risk pregnancies and receives referrals for complicated pregnancies from other sectors at all gestations. Antenatal women routinely have an antenatal booking Hb measured between eight and 16 weeks, depending on whether they opt for genetic screening along with an Hb electrophoresis if their thalassemia status is unknown. All pregnant women receive folic acid 5 mg daily until 12 weeks and a multivitamin preparation thereafter. The incidence of anemia in the first trimester in the Singapore population is 22.2% [18], with the vast majority (72%) due to iron deficiency [19], with an estimated 9% incidence of thalassemia in our population [20]. The Hb level is then repeated between 24 and 28 weeks at the time of an oral glucose tolerance test (GTT).

For patients diagnosed with anemia (Hb <10.5 g/dL) at 24-28 weeks, serum ferritin is performed. Women diagnosed with iron deficiency anemia are offered oral iron supplementation. Daily morning oral iron supplementation of 40-80 mg is offered and continued for three months or six weeks postpartum.

Since 2021, we have begun offering intravenous ferric carboxymaltose administration as a day procedure in our Obstetric Day Assessment Centre (ODAC). Intravenous ferric carboxymaltose (Ferinject 1,000 mg in 20 mL) is available as a treatment for patients with an Hb of <10.5 g/dL without risk of iron overload; have a ferritin level of <30 μg/L; are intolerant, refractory, or noncompliant with oral iron treatment; presenting with symptomatic anemia; or are at an advanced gestation >34 weeks. Intravenous ferric carboxymaltose is contraindicated in patients with active infection, in their first trimester of pregnancy, those with hepatic impairment, severe asthma/eczema/atopy, allergy to intravenous iron, non-iron deficiency anemia, or at risk of iron overload.

Blood transfusion is reserved for patients with severe active bleeding, imminent cardiac compromise, or symptoms of anemia requiring urgent attention.

Study design

This was a retrospective study looking at the outcomes, in terms of changes in hematological maternal parameters and obstetric outcomes, of 127 women who were referred for outpatient intravenous ferric carboxymaltose administration at our obstetric day assessment between July 2021 to May 2023. Results of antenatal patients who received intravenous ferric carboxymaltose (case group) were compared to those who met the referral criteria but defaulted on treatment (control group).

Ethical considerations

Waiver of consent was approved by our Institutional Review Board per the guidelines (SingHealth Central Institutional Review Board reference number 2023/2375).

Study criteria

All patients who were referred for intravenous ferric carboxymaltose administration at ODAC between July 2021 to May 2023 were included in our study. The case group comprised 109 patients who received intravenous ferric carboxymaltose, and the control group comprised 18 patients who met the criteria for intravenous ferric carboxymaltose administration but defaulted on or declined treatment. We compared these groups as they had similar characteristics meeting the criteria for outpatient treatment with intravenous ferric carboxymaltose and were both treated with oral iron supplements. However, those in the case group received intravenous ferric carboxymaltose, whereas those in the control group did not. Because of the low number of patients who defaulted on their appointments and did not receive intravenous ferric carboxymaltose, the control group was smaller than the case group. 

Procedure and assessment

Retrospective data of patients who were referred for intravenous ferric carboxymaltose administration at our obstetric day assessment unit during our study period were collected, including demographics, hematological maternal parameters, and outcomes of pregnancy.

Patients who were referred for treatment but did not receive it were included in the control group, while those who received the treatment were included in the case group. Because of the low number of patients who defaulted on their appointments and did not receive intravenous ferric carboxymaltose, the control group was smaller than the case group.

This is a case-control study where we compared the results at delivery of patients who received intravenous ferric carboxymaltose in the case group compared to those in the control group who did not receive the treatment. The patients in the case and control groups shared similar characteristics and met the criteria for referral for outpatient treatment with intravenous ferric carboxymaltose. The outcomes at delivery among those who received ferric carboxymaltose were compared to those who did not in order to compare the efficacy of treatment.

Case-control design

We studied the obstetric and hematological outcomes of obstetric patients diagnosed with iron deficiency anemia who received intravenous ferric carboxymaltose in the case group compared to the control group who did not. Both groups met the criteria for referral for intravenous ferric carboxymaltose at our center, and they were treated with oral iron supplements and had similar characteristics. Patients in the case group received intravenous ferric carboxymaltose, while those in the control group did not. This allows us to study the effects and side effects of intravenous ferric carboxymaltose administration in pregnancy.

Sample size calculation

This is a retrospective study looking at our outcomes after initiation of outpatient intravenous ferric carboxymaltose treatment; therefore, all cases within the study period were included. Further studies can be considered.

Statistical analysis

Statistical analysis was performed via Statistical Product and Service Solutions (SPSS) (version 23; IBM SPSS Statistics for Windows, Armonk, NY) through a paired t-test.

## Results

A total of 127 obstetric patients were referred for outpatient intravenous ferric carboxymaltose treatment between 2021 and May 2023. Of these patients, seven were twin pregnancies, and 120 were singleton pregnancies. Eighteen patients defaulted on their appointments. The results of 109 patients who received intravenous ferric carboxymaltose (case group) were compared to those of the 18 patients who defaulted (control group). The maternal age of our patients ranged from 18 to 42 years old, with a mean age of 31.2 years old. Of these patients, 24 were Chinese, 84 were Malay, 17 were Indian, and two were of other ethnicities. Approximately 49 patients were primiparous, and 78 were multiparous with two to nine children (inclusive of current pregnancy). Approximately 43 patients had serum vitamin B12 and folate levels checked as part of an anemia workup, of which seven had vitamin B12 deficiency and eight had folate deficiency. These patients were treated accordingly with vitamin B12 and oral folic acid. All patients were treated with oral iron therapy. Five patients had antenatal transfusion because of symptomatic anemia or low Hb <8 g/dL despite iron therapy. We collected data on gestational age at booking, Hb and mean corpuscular volume (MCV) at booking, and those on the second/third trimesters. Serum ferritin, Hb, and MCV post ferric carboxymaltose, as well as Hb and MCV at delivery, were also analyzed.

Demographics including age, parity, ethnicity, presence of thalassemia trait, and serum ferritin levels were compared between the case and control groups and were similar. In the case group, the median gestational age at booking was 11.7 weeks, and the median Hb at booking was 10.6 g/dL with a median MCV of 76.2 fL. In the routine FBC performed in 24-28 weeks, the median Hb in the case group has dropped to 8.9 g/dL with a median MCV of 76.7 fL. All the patients in the case group were treated with oral iron supplementation with either iron polymaltose (Maltofer 100 mg twice daily) or ferrous gluconate (Sangobion, two tablets daily). Hb and MCV did not significantly increase after oral treatment in our case group, with median Hb of 8.5 g/dL and MCV of 75.5 fL at 33.4 weeks (range 14.0-39.3 weeks). These patients were then referred for intravenous ferric carboxymaltose treatment in the outpatient setting. Two patients in the case group reported adverse side effects after completion of ferric carboxymaltose treatment including nausea/vomiting in one patient and giddiness in the other patient. Both patients were monitored post procedure in ODAC and discharged after observation as both remained well, and symptoms had resolved. None of the patients in the case group had to be hospitalized or received any additional treatment because of ferric carboxymaltose administration.

All obstetric patients at our center routinely have an FBC including Hb and MCV taken at admission during delivery. Pregnancy outcomes including gestational age, Hb, MCV, fetal weight, mode of delivery, and any antenatal transfusion were compared between the case and control groups.

Data were analyzed using SPSS through a paired t-test and one-way ANOVA. A P value of <0.05 was taken to be significant. 

Table 1 shows the demographics and investigation results of the patients included in this study. The case group included patients who received intravenous ferric carboxymaltose, whereas the control group included patients who defaulted on their appointments and did not receive intravenous ferric carboxymaltose.

**Table 1:** Patient demographics Hb: hemoglobin; MCV: mean corpuscular volume. Statistical parameters wherein data are measured are represented in the left-sided columns accordingly. A P value of <0.05 is considered as significant. *Data are shown as median (first quartile to third quartile) or n (%). †Treatment-related adverse outcomes included nausea, vomiting, and giddiness.

Characteristics*	Case group (n=109)	Control group (n=18)
Age, years	31 (27.5-35.0)	32.5 (27.5-37.0)
Parity, n	2 (1-3)	2 (1-3)
Chinese (%)	19 (14.9)	5 (1.8)
Malay (%)	72 (56.7)	12 (4.3)
Indian (%)	16 (12.6)	1 (0.5)
Others (%)	2 (1.6)	0 (0.0)
Singleton pregnancy (%)	103 (81.1)	17 (13.4)
Twin pregnancy (%)	6 (4.7)	1 (0.8)
Thalassemia trait (%)	14 (11.0)	4 (3.1)
No thalassemia trait (%)	95 (74.8)	14 (11.0)
Serum ferritin, μg/L	7.75 (5.8-12.3)	9.0 (5.8-13.0)
Gestational age at booking, weeks	11.7 (8.0-12.0)	11.2 (6.4-28.7)
Hb at booking, g/dL	10.6 (9.3-11.3)	10.8 (9.2-11.6)
MCV at booking, fL	76.2 (69.3-82.9)	78.2 (70.7-84.4)
Gestational age at Hb/MCV in second/third trimester, weeks	25.4 (22.4-32.1)	25.7 (23.9-32.7)
Hb in second/third trimester, g/dL	8.9 (8.3-9.5)	9.25 (8.3-9.6)
Difference in Hb in second/third trimester and at booking, g/dL	-1.5 (-2.8-2.3)	-1.4 (-2.5-0.5)
MCV in second/third trimester, fL	76.7 (70.9-80.0)	80.2 (70.7-84.3)
Difference in MCV at second/third trimester and at booking, fL	1.1 (-4.0-2.5)	2.7 (-3.7-2.9)
Gestational age at repeated Hb/MCV post iron supplementation, weeks	33.4 (25.0-39.0)	33.2 (26.9-39.4)
Hb post oral iron supplementation, g/dL	8.5 (8.0-9.2)	8.8 (7.9-9.1)
Difference in Hb post iron supplementation and in second/third trimester, g/dL	-0.1 (-1.2-0.4)	-0.25 (-1.6-0.5)
MCV post oral iron supplementation, fL	75.5 (70.9-80)	74.1 (70.7-84.3)
Difference in MCV post iron supplementation and in second/third trimester, fL	-0.7 (-1.5-1.2)	-1.0 (-1.8-1.6)
Gestational age at ferric carboxymaltose treatment, weeks	33.4 (29.0-35.0)	-
Treatment-related adverse outcomes reported	2^# ^(1.6%)	-

The number of patients in the control group was 18 compared to 109 in the case group. The mismatch between the number of patients in each group is because of the low defaulter rate, as most patients were compliant with treatment as advised by their clinicians.

Table 1 shows that the median Hb at booking was 10.6-10.8 g/dL, which dropped to 8.9-9.2 g/dL in the second/third trimester (based on the routine antenatal FBC performed at 24-28 weeks gestation). Hb levels dropped by 1.5 g/dL in the case group and 1.4 g/dL in the control group. Despite four weeks of oral iron supplementation, Hb remained at 8.5-8.8 g/dL, with a 0.2-0.3 g/dL decrease compared to the Hb levels before iron supplementation.

Moreover, we looked at the pregnancy outcomes in both groups after treatment with intravenous ferric carboxymaltose, including Hb at delivery, mode of delivery, fetal weight, and need for antenatal transfusion.

Table 2 shows the pregnancy outcomes in the case and control groups.

**Table 2:** Pregnancy outcomes in cases and controls Hb: hemoglobin; MCV: mean corpuscular volume. Statistical parameters wherein data are measured are represented in the left-sided columns accordingly. A P value of <0.05 is considered as significant. *Data are shown as median (first quartile to third quartile) or n (%).

Pregnancy outcome*	Case group (n=109)	Control (n=18)	P value
Gestational age at delivery, weeks	38.7 (37.0-39.0)	38.7 (37.0-39.0)	0.374
Hb at delivery, g/dL	10.7 (9.7-11.6)	8.9 (9.2-11.3)	0.000
Hb at delivery ≥9 (%)	103 (94.0)	9 (50.0)	
Hb at delivery ≥10 g/dL (%)	80 (73.4)	5 (27.8)	
Difference between Hb at delivery and post-oral iron supplementation, g/dL	2.3 (0.6-2.9)	0.35 (-0.3-3.0)	0.000
MCV at delivery, fL	82.2 (77.0-86.3)	73.0 (75.5-87.8)	0.056
Difference between MCV at delivery and post-oral iron supplementation, fL	6.0 (1.8-9.7)	0.0 (-0.3-10.2)	0.000
Singleton babies’ fetal weight, g	3,101 (2,737.5-3,287.8)	2,910 (2,737.5-3,287.8)	0.906
Twin babies' fetal weight, g	2,282 (1,640-2,854)	950 (950-1,000)	0.330
Spontaneous vaginal delivery	63 (57.8%)	10 (55.6%)	0.977
Assisted vaginal delivery	7 (6.4%)	2 (11.1%)	
Elective cesarean delivery	16 (14.7%)	2 (11.1%)	
Emergency cesarean delivery	23 (21.1%)	4 (22.2%)	
Antenatal transfusion	8 (7.3%)	1 (5.6%)	0.787
No antenatal transfusion	101 (92.7%)	17 (94.4%)	

Table 2 shows that the median Hb at delivery was 10.7 g/dL compared to 8.9 g/dL in the control group. Hb at the time of delivery increased by 2.3 g/dL (median) in the case group compared to only 0.35 g/dL (median) in the control group.

Our results showed that there was an overall decrease in Hb from booking to second/third trimesters. The decrease in Hb from booking to the second/third trimester was 1.5 g/dL in the case group and 1.4 g/dL in the control group (median). Despite oral iron supplementation, the median Hb did not increase antenatally in both the case and control groups, with A 0.1 g/dL decrease in the case group and 0.25 g/dL in the control group (median). MCV also did not improve after iron supplementation in either the case or control groups, with a decrease of 0.7 dL in the case group and 1.0 fL in the control group (median).

Comparing the case group who received intravenous ferric carboxymaltose and the control group who did not, there were significant differences in the Hb at delivery (10.7 g/dL compared to 8.9 g/dL, P value 0.000). The difference in Hb at delivery compared to post-iron supplementation was 2.3 g/dL in the case group and 0.6 g/dL in the control group (median, P value 0.000). Moreover, there was a significant difference in MCV at delivery compared to post-iron supplementation with a 6.0 fL increase in the case group and a 0.3 fL increase in the control group (median, P value 0.000). 

However, the increase in Hb did not translate to a difference in pregnancy outcomes in terms of gestational age at birth, fetal weight, mode of delivery, or the need for antenatal transfusion. As seen in Table 2, the administration of intravenous ferric carboxymaltose did not show any significant differences in the gestational age at birth, fetal weight, and mode of delivery. The need for antenatal transfusion did not significantly differ between the two groups. The need for antenatal blood transfusion was 8.0% (8/109) in the case group and 6.2% (1/18) in the control group, and this result was not statistically significant. Antenatal blood transfusion was given to one patient in the control group as her Hb at delivery was less than 8.0 g/dL. In the case group, eight patients required antenatal transfusion. The reason for antenatal transfusion was because of a low booking Hb of 5.3 g/dL in one patient, second-/third-trimester Hb less than 8.0 g/dL in six patients, and symptomatic anemia with a Hb of 8.2 g/dL in one patient.

## Discussion

There have been several previous studies on the safety and efficacy of ferric carboxymaltose in pregnancy [21-23]. In a case-control study over two years in the Netherlands, Pels et al. found a mean rise in Hb of 2.3 g/dL in 64 women treated with ferric carboxymaltose at an average gestational age of 34+6 weeks from 8.4 g/dL to 10.7 g/dL at term, which was comparable to 64 non anemic controls [21]. There were no adverse side effects in the treatment group in this study. A similar rise in Hb at four weeks was found in 44 patients treated with ferric carboxymaltose in a comparative study with CosmoFer (iron dextran). In this study, the Hb increment increased with time after treatment, and it was higher for ferric carboxymaltose at two and four weeks but slightly lower at six weeks compared with CosmoFer. No adverse events were reported in either arm of the study [24]. Qassim et al. in a retrospective cohort study found that intravenous iron supplementation was effective in improving maternal hematological parameters and reducing iron deficiency anemia during delivery and, compared to oral iron, produced a greater rise in Hb at delivery [25].

A recent open-label randomized controlled trial compared 436 women with Hb <10 g/dL at 13-26 weeks given 1 g intravenous ferric carboxymaltose against 432 women given 60 mg elemental iron [26]. The rate of anemia was numerically lower in the ferric carboxymaltose group than for those receiving oral iron at all subsequent gestational ages. There was no statistical difference in the birthweights of the infants in both groups/ferric carboxymaltose appears to improve ferritin at term, which, in combination with the physiological dieresis, might speed up the restoration of the Hb level postnatally.

Studies comparing the adverse effects of ferric carboxymaltose to oral iron also showed that intravenous ferric carboxymaltose had a lower rate of complications [27] and produced less constipation, nausea, and vomiting as well as diarrhea [28].

Our study demonstrates the safety of treating women with intravenous iron supplementation in an outpatient setting in centers with adequate manpower and equipment to support the procedure. The incidence of side effects among patients who received ferric carboxymaltose was low in our study (2/127, 1.6%), and the side effects experienced by patients were both mild and did not require further medical therapy or admission. The acceptance rates among patients receiving ferric carboxymaltose in an outpatient setting were high because 109/127 (85.9%) of patients who were referred for outpatient ferric carboxymaltose administration attended their appointments.

From the results of our study, there was a significant decrease in Hb in the second/third trimester compared to booking blood (mean decrease of 1.4 g/dL in both case and control groups) in our obstetric patients. Despite oral iron supplementation, both groups did not experience any significant improvement in Hb (-0.1 g/dL and -0.3 g/dL) and were referred for intravenous ferric carboxymaltose treatment.

Our results suggest a significant rise in Hb (2.3 g/dL compared to 0.6 g/dL, P value=0.00) and MCV (6.0 fL compared to 0.3 fL, P value=0.00) post ferric carboxymaltose administration in the case group compared to those in the control group at delivery. Hb at delivery was significantly higher among the case group who received intravenous ferric carboxymaltose compared to the control group who did not (10.7 g/dL compared to 8.9 g/dL, P value=0.00).

Previous studies have demonstrated that maternal anemia is associated with maternal and fetal complications including preterm labor, low birthweights, and antenatal blood transfusion [29,30]. However, gestational age at delivery, fetal weight, mode of delivery, and risk of antenatal transfusion did not differ significantly between the two groups in our study, despite the overall increase in Hb at delivery after antenatal intravenous ferric carboxymaltose administration.

The main advantage of intravenous ferric carboxymaltose administration is to increase the Hb before delivery, particularly among patients who did not respond, were unable to tolerate, or were noncompliant with oral iron supplementation. This may potentially decrease the rate of postnatal transfusion in patients with iron deficiency anemia who experienced further blood loss during delivery.

We acknowledge that our study has its limitations. Data comparing the serum ferritin at booking and delivery were not collected as these were not part of the routine antenatal protocol in our center. Patient compliance with oral iron supplements is difficult to ascertain and measure and, therefore, was not included in the analysis; however, this could be a confounder. The difference in the number of patients between the case and control arms because of the low defaulter rate may also affect our results. Further studies can be performed to study the safety and efficacy of antenatal intravenous ferric carboxymaltose treatment among obstetric patients with iron deficiency anemia as well as obstetric outcomes after treatment of iron deficiency anemia in these patients.

## Conclusions

This study examines obstetric patients from a multiethnic Southeast Asian obstetric population, and it contributes to the literature and highlights areas in need of further study concerning the safety and efficacy of intravenous iron supplementation in the obstetric population. This is particularly important because of the high percentage of obstetric patients with iron deficiency anemia worldwide.

In conclusion, intravenous ferric carboxymaltose is a safe and effective treatment for obstetric patients with iron deficiency anemia who do not respond or are noncompliant with oral iron supplementation. It can be given as an outpatient therapy and has a high acceptance rate among patients. A single dose of intravenous ferric carboxymaltose injection antenatally can significantly increase Hb and MCV at delivery and decrease the incidence of maternal iron deficiency anemia. This may decrease the need for intrapartum and postpartum blood transfusion, which carries with it risks of blood transfusion reactions, transmission of infections, and other complications. Further larger studies comparing the effects of intravenous and oral dosing, examining any maternal and neonatal benefits and complications, and looking at the effects on rates of perinatal blood transfusion are required.
